# Anxiety characteristics in benign paroxysmal positional vertigo: first vs. recurrent episodes

**DOI:** 10.1007/s00405-024-08615-y

**Published:** 2024-04-04

**Authors:** Lotem Mann Ben Yehuda, David Rachima, Michal Katz-Leurer

**Affiliations:** 1 Physical Therapy Department, Faculty of Medicine, School of Health Professions, Tel-Aviv University, Tel-Aviv, Israel; 2 Maccabi Health Care, Kfar Saba, Israel; 3 Maccabi Health Care, Netanya, Israel

**Keywords:** Anxiety, Benign paroxysmal positional vertigo, Episode, Recurrence

## Abstract

**Purpose:**

To assess the difference in state and trait anxiety levels in patients with Benign Paroxysmal Positional Vertigo (BPPV) at the first episode (FE) versus recurrent episodes (RE), before and after vestibular physiotherapy. A secondary objective was to assess the difference in the prevalence of underlying health conditions between FE and RE BPPV patients.

**Methods:**

Fifty-five patients with BPPV, aged 40–70, were recruited. The diagnosis of BPPV was confirmed based on subjective complaints of vertigo and positive results from the Dix–Hallpike and Supine Roll tests. Twenty-four patients were in their FE, and 31 had RE. All patients completed the State–Trait Anxiety Inventory (STAI) questionnaire three times; before, immediately after, and a week after vestibular physiotherapy treatment.

**Results:**

The RE group demonstrated higher trait anxiety than the FE group in all testing points: before treatment (median value of 38 versus 29, *p*-value = 0.02), immediately after treatment (median value of 36 versus 28, *p*-value < 0.01) and a week later (median value of 38 versus 28, *p*-value < 0.01). State anxiety decreased immediately after treatment in both groups, but at the second session, it was significantly higher in the RE than in the FE group (median value of 38 versus 28.5, *p*-value = 0.03). Hypothyroidism was significantly more frequent in the RE group (RE 16%, FE 0%, *p*-value = 0.04).

**Conclusions:**

Based on the current study’s findings, we recommend assessing anxiety levels in patients with recurrent BPPV and consider referring them for appropriate treatment when necessary.

## Introduction

Benign paroxysmal positional vertigo (BPPV) is the most common disorder of the vestibular system [1], with a lifetime prevalence rate of 2.4% [2]. BPPV is characterized by rotational vertigo episodes that occur when the head’s position is changed in relation to gravity. In about 20% of cases the disorder passes spontaneously within a month and in about 50%, within 3 months [2, 3]. Although BPPV generally responds well to vestibular treatment, there is a high recurrence rate after successful treatment. The recurrence rate is approximately 10–30% during a 1-year follow-up period and 10-year recurrence could be as high as 50% [4, 5].

Several studies have demonstrated that various pathological conditions may be associated with recurrence of BPPV [6]. Female gender, cardiovascular diseases (hypertension, diabetes mellitus, hyperlipidemia) as well as osteoporosis and vitamin D deficiency are risk factors for recurrent BPPV [7]. Association with recurrence is stronger when multiple diseases act synergically [3, 8]. The most frequently involved pathologies are psychiatric disorders, followed by neurological and vascular diseases [9]. In addition, physical inactivity was found to be associated with BPPV episodes (Risk ratio = 2.6) [8].

It is well-established that dizziness is a stressful experience that can increase fear and anxiety [10]. A recent review study found that patients with BPPV are 3.19 times more prone to have anxiety disorders compared to controls [11]. The anticipatory fear of acute and unexpected vertigo may negatively affect patients with BPPV and increase their anxiety levels regardless of whether they previously suffered from this condition [10]. On the other hand, evidence points to a reversed relationship: a prospective historical study found that anxiety disorder patients may have an increased risk of developing BPPV (2.2 times higher than among patients without anxiety) [12].

If the anxiety experienced by the patients is temporary and not their personality trait, the anxiety caused by BPPV is mainly a condition [13]. If this anxiety decreases after successful treatment (success rate of about 80% after one treatment and 92% after second treatment) [3], then the anxiety as a contributing factor to the recurrence of BPPV is questionable.

The primary aim of this study is to assess the difference in state and trait anxiety between patients with a first episode (FE) versus a recurrent episode (RE) of BPPV before and after vestibular physiotherapy. A secondary aim is to assess the difference in underlying health conditions between BPPV patients with FE and RE.

## Methods

### Participants

Included were patients between ages 40–70 with BPPV. The diagnosis of BPPV was confirmed by the subjective complaint of a spinning sensation (vertigo) elicited by head motion relative to gravity (such as when changing position in bed or bending over), and an objective positive Dix–Hallpike test or Supine Roll test (presence of positional nystagmus) [2]. Additionally, based on patients’ self-report of BPPV history, they were divided into two groups: the FE group, consisting of 24 patients, and the RE group, consisting of 31 patients who suffered from at least one episode of BPPV, with at least a month passing since the last episode.

Excluded were patients who currently or in the past suffered from the following vestibular problems: vestibular migraine, vestibular neuritis, vestibular labyrinthitis, Meniere’s disease, central positional vertigo, perilymphatic fistula, superior canal dehiscence syndrome, or who had undergone ear surgery. In addition, patients suffering from other medical conditions contraindicated for vestibular physiotherapy treatment and those who do not speak Hebrew were excluded.

The study took place in a public outpatient physiotherapy clinic (Maccabi Health Care) in Kefar-Saba, Israel.

## Tests and measurements

### Outcome measures.

#### The State–Trait Anxiety Inventory (STAI)

A self-administered questionnaire with two parts to separately assess the level of state and trait anxiety. Developed by Spielberger [14] and translated into Hebrew [15]**,** each part includes 20 items, and the score varies from 20 to 80 points. The higher the score, the higher the anxiety, with a cut-off score of 39–40 that clinically reflects anxiety [16]. The questionnaire demonstrates a high internal consistency [17] and has been validated against the Taylor Manifest Scale and Cattell and Scheier’s Questionnaire [16].

#### Dizziness handicap inventory (DHI)

A self-administered questionnaire is used to quantify the effect of dizziness on day-to-day functioning and the perception of self-disability. The DHI questionnaire includes 25 items classified into three subgroups: physical, functional and emotional aspects of dizziness. It is a practical and accepted tool in the clinic to evaluate the function, quality of life, and changes in these aspects after therapeutic intervention in patients with vertigo [18, 19]. The Hebrew version of the DHI questionnaire was used [10]. The DHI questionnaire was validated against the short form-36 function questionnaire, and showed a moderate to high criterion validity [20].

### Screening and background measures

The diagnosis of BPPV was confirmed by the subjective complaint of a spinning sensation (vertigo) elicited by head motion relative to gravity and a positive Dix–Hallpike test (HD) and Supine Roll test (SRT)—presence of positional nystagmus [2].

Medical history, demographics, and characteristics of the BPPV episode were obtained and confirmed from the patients’ self-reports and medical files. The Godin–Shephard, a self-administered physical activity questionnaire, was used to quantify the intensity and duration of average physical activity in a typical week [21].

### Procedure

Patients with BPPV recommended by their physicians for referral to vestibular physiotherapy evaluation and treatment, were offered to participate in the study. A certified vestibular physiotherapist performed the study during two sessions. The study was approved by the Helsinki Committee of the Maccabi Health Care Services and the Ethics Committee of Tel Aviv University. Written informed consent was obtained from all patients. After signing the informed consent form, patients were asked to complete the STAI and DHI questionnaires. Patients’ demographics, medical history, and characteristics of symptoms were recorded. Afterwards, the HD and SRT diagnostic screening tests were performed. The examiner carefully observed the patients’ eyes to detect nystagmus [2]. If no evidence of nystagmus was found, patients with suspected BPPV were excluded from the study. Patients with BPPV were treated with the appropriate Canalith Repositioning Procedure (e.g., Epley maneuver), based on the Clinical Practice Guidelines for assessing and treating BPPV. In the case of positive BPPV signs in two canals, a maneuver was performed on the canal where the vertigo was more significant [2]. At the end of the treatment, all patients were asked to complete the STAI and Godin–Shephard physical activity questionnaires.

A week later, the patients arrived for a second evaluation where they filled out the STAI and DHI questionnaires. After that, another HD and SRT test was performed to assess the need for further treatment Based on these test results, each group (FE and SE) was divided into two subgroups: presence vs absence of positive BPPV signs in the second session.

### Statistical methods

Nonparametric statistic tests were used. Differences between groups were tested using the *χ*^2^ and Whitney–Mann tests. The Wilcoxon test assessed differences in outcome measures following treatment or between sessions.

Spearman’s correlation coefficient test was used to examine the relationship between the number of BPPV episodes in the RE group and the levels of anxiety.

To test the differences between the subgroups according to the group’s division (FE vs. RE) and positive BPPV signs in a second meeting, the change in anxiety levels was analyzed using the Kruskal–Wallis test, combined with the Wilcoxon test with Bonferroni’s first-type error correction. Statistical analysis was performed with IBM SPSS Statistics Software (version 27). Statistical significance was defined as a *p*-value < 0.05.

## Results

Table 1 summarizes the patients’ demographic and health characteristics divided into groups.

**Table 1:** Demographic data, level of physical activity, and background health characteristics by groups

	First episode (*N* = 24)	Recurrent episodes (*N* = 31)	*p*-value
Age (years)	55 [44–70]	559 [41–70]	0.59
Gender (female)	18 (75.0)	25 (80.6)	0.62
Godin and Shephard (units)	51 [0–252]	30 [0–168]	0.13
Background health characteristics			
High blood pressure	5 (21)	8 (26)	0.67
Diabetes	3 (13)	7 (23)	0.34
High cholesterol	6 (25)	8 (26)	0.95
Osteoporosis	3 (13)	5 (16)	0.71
Vitamin D deficiency	15 (63)	15 (48)	0.3
Migraines	0 (0)	1 (3)	0.38
Head injury	2 (8)	0 (0)	0.1
Heart or blood vessel problems	3 (13)	1 (3)	0.19
Neurological diseases	0 (0)	1 (3)	0.38
Autoimmune diseases	1(4)	1 (3)	0.85
Hypothyroidism	0 (0)	5 (16)	0.04
Total number	1 [0–6]	1 [0–5]	0.67

Values in table are median [minimum–maximum] or number (percentage), *p*-value is based on Mann–Whitney *U* test or *χ*^2^

Patients’ median age was 57 years (range 41–70 years), and 78% were women, with non-significant differences between groups. No significant difference was found between the groups in their health characteristics, except that in the RE group, the frequency of hypothyroidism is five women (16%), compared to zero women/men in the FE group (*p* = 0.04).

Table 2 summarizes the BPPV characteristics and DHI questionnaire scores. Approximately 93% of all patients presented with an impairment in the posterior canal, with no significant difference between groups. Among the patients with RE of BPPV, ten (32%) experienced a second episode, 13 (42%) experienced three to five episodes, and eight (26%) had six or more episodes.

**Table 2:** The semicircular canal affected and dizziness handicap inventory values in the first and second sessions by groups

First session	First episode (*N* = 24)	Recurrent episodes (*N* = 31)	*p*-value
Semicircular canal			
Posterior right	9 (37.5)	12 (38.7)	0.93
Posterior left	10 (41.7)	16 (51.6)	0.46
Horizontal right	1 (4.2)	1 (3.2)	0.85
Horizontal left	1 (4.2)	0 (0)	0.25
Multi-canal	3 (12.5)	2 (6.5)	0.44
Dizziness handicap inventory (/100)	47 [2–80]	38 [8–99]	0.42

Second session	*N* = 22	*N* = 27	
Positive BPPV test	15 (62.5)	11 (35.5)	0.06
Dizziness handicap inventory (/100)	25 [0–66]	22 [0–76]	0.98

Values in table are median [minimum–maximum] or number (percentage), *p*-value is based on Mann–Whitney *U* test or *χ*^2^

Only 22 patients from the FE group and 27 from the RE group attended the second session. No significant difference was found between the groups in the DHI values in the first and second sessions. However, a significant decrease was observed in each group in the DHI values between sessions, with a reduction of a median value of 6 points in the FE group (*p*-value = 0.01) and a decrease of a median value of 12 points in the RE group (*p*-value < 0.01).

Table 3 summarizes the difference in trait and state anxiety between groups before and after vestibular physiotherapy. No significant difference was found in the state anxiety score between the groups before the first treatment (FE = 44, RE = 49, *p*-value = 0.1). Both groups showed a decrease in values following treatment in the first session (pre–post treatment) and between the first and second sessions. However, in the second session, the median values of state anxiety in the RE group were 9.5 points higher than in the FE group, and this difference was significant.

**Table 3:** State and trait anxiety levels according to the State–Trait Anxiety Inventory questionnaire in the study stages, stratified by group

	First episode (*N* = 24)	Recurrent episodes (*N* = 31)	*p*-value^#^
**State anxiety**			
Pre treatment	44 [20–63]	49 [23–82]	0.1
Post-treatment	36 [20–64]	35 [24–62]	0.83
*p*-value*	0.01	< 0.01	
Second session	28.5 [18–50]	38 [21–72]	0.03
*p*-value*	< 0.01	< 0.01	
**Trait anxiety**			
Pre treatment	31 [22–52]	38 [21–58]	0.02
Post-treatment	29 [21–48]	36 [21–64]	0.02
*p*-value*	0.02	0.08	
Second session	28 [20–49]	38 [24–58]	< 0.01
*p*-value*	0.01	0.99	

Values in table are median [minimum–maximum], ^#^*p*-value is based on Mann–Whitney *U* test, *p-value is based on Wilcoxon

In addition, among all the patients, a moderately strong and positive association (rs = 0.5) was found between DHI and state anxiety level before treatment in the first session (*p*-value < 0.001) and in the second session (rs = 0.66) (*p*-value < 0.001). These data are not presented in the table.

The patients in the RE group showed higher trait anxiety values at all test points. In the RE group, no significant difference was found when comparing the values of trait anxiety before and after treatment and between the first and second sessions. In the FE group, trait anxiety values decreased significantly between the initial evaluation and the two subsequent evaluations (31 [22–52] to 29 [21–48] and 28 [20–49], respectively, *p*-value = 0.02 and 0.01).

In the RE group, no association was found between the number of BPPV episodes and the levels of trait anxiety.

In the second session, among patients with a FE, 62.5% had positive BPPV signs compared to 35.5% in the RE group, with a borderline significant difference between the groups (*p*-value = 0.06).

**Fig. 1:**
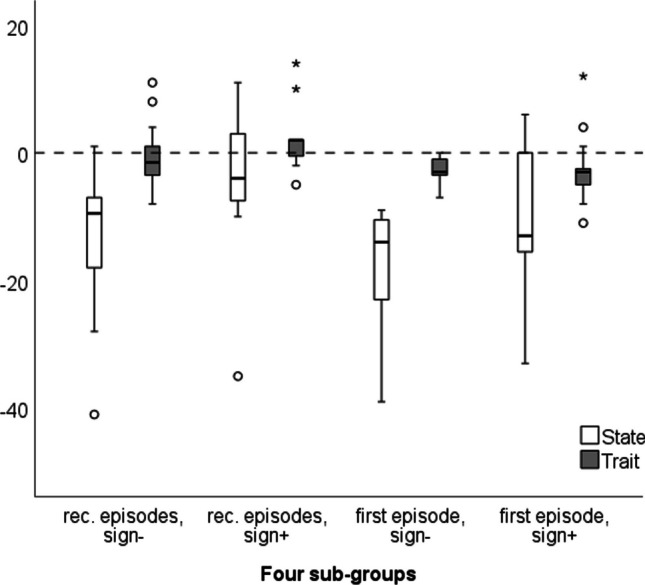
The difference in the absolute values of the state and trait anxiety indices between the first meeting and the second, divided into subgroups

Figure 1 shows the difference in the absolute values of the state and trait anxiety indices between meetings, divided into four subgroups: FE vs. RE, each split into non vs. positive BPPV signs in the second meeting.

It was found that there is a significant difference between the four subgroups in the degree of change in state anxiety (*p*-value = 0.027) and trait anxiety (*p*-value = 0.024). Post hoc analysis reveals that the source of the difference between groups is a significant difference between the FE without BPPV signs in the second session (median decrease value of 14 in the state score and 3 points in the trait score) as compared to patients with RE and positive BPPV signs in the second session (median value of 4 points and -2, respectively).

## Discussion

A high prevalence rate of anxiety in people with BPPV and, conversely, a high prevalence rate of BPPV in people with anxiety, have been well described in the literature [4, 12]. Previous studies have shown a decrease in anxiety following BPPV treatment [22, 23]. This study adds to what is known by focusing on the nature of the anxiety.

The main finding of this study is that patients with RE BPPV have a significantly higher trait anxiety level than those with FE BPPV, and this was consistent throughout both sessions. In the FE group, trait anxiety values showed a small reduction (2–3 points) between the initial evaluation and the subsequent two evaluations. Although the change was statistically significant (*p* = 0.02/0.01), it does not appear to represent a clinically meaningful change.

In addition, the baseline state anxiety levels were high in both groups with no significant difference between them. However, in both groups, a significant reduction was noted in state anxiety between the first and the second sessions. However, in the second session Interestingly, patients with RE who showed positive BPPV signs at the second meeting did not experience improvements in state anxiety as did all other subgroups.

Dizziness is a stressful experience that can increase fear and anxiety [4]. It has been observed that the success rate of physiotherapy treatment for BPPV is high, with approximately an 80% success rate after the first treatment [3]. Successful treatment is linked with a decrease in dizziness, as well as with the associated fear and anxiety. The current study underscores that this reduction is manifested predominantly in state anxiety.

In addition, the results of this study reinforce the assumption that individuals with RE BPPV tend to experience higher levels of anxiety compared to those with FE BPPV, as evidenced by elevated trait anxiety levels during both sessions. A recent study demonstrated that anxiety-depression status significantly reduces the efficacy of first treatment and increases the risk of recurrence in BPPV [24]. Furthermore, the current study’s results indicate that patients with RE who exhibit positive BPPV signs at the second meeting do not experience improvement in either state or trait anxiety levels. Previous studies offer some possible explanations. First, anxiety and depression are associated with a chronic low-grade inflammatory response. This may increase oxidative and nitrosative stress and exacerbate vestibular degeneration, consequently leading to difficulty with the relocation and reabsorption of the dislodged otoliths [5, 24]. Second, anxiety causes a neuroendocrine response dysfunction (abnormal activation of the hypothalamus–pituitary–adrenal axis) that changes the blood flow of the inner ear and interferes with the recovery of BPPV [5]. Third, there is a link between the vestibular and emotional processing systems. Neurotransmitters and neuroanatomical regions involved in the vestibular systems and emotional responses are similar. The vestibular nuclei are connected through the parabrachial nuclei to the brain regions responsible for the response to fear and anxiety; both areas affect the autonomic system [11, 25].

Previous studies have emphasized the importance of assessing anxiety in individuals with BPPV [5, 11, 12, 24, 26]. The current study highlights the need to evaluate anxiety, particularly trait anxiety, in the RE group. Moreover, it would be valuable to conduct a follow-up study among patients after their FE to assess whether higher levels of state anxiety and positive BPPV signs at the second meeting are more indicative of developing recurrent BPPV events.

The only health characteristics that were significantly different between groups was the frequency of hypothyroidism which was present in five women (16%), in the RE group compared to zero women/men in the FE group. This is in line with findings in previous works [27, 28]. Hypothyroidism is found to be associated with ear-related deficits such as hearing loss, tinnitus, and vestibular deficits [27], and patients with RE have a higher incidence of hypothyroidism than patients with FE [28]. Researchers have proposed that the connection between hypothyroidism and BPPV may be related to changes in the fluid composition in the ear due to hormonal changes, which disrupt the function of the sodium channels. Additionally, low thyroid function is associated with cardiovascular changes and blood pressure alterations that reduce blood flow to the inner ear. Both factors can affect the release and absorption of otoconia [28].

## Limitations

A limitation of this study is the relatively small number of patients who had more than two episodes of BPPV compared to the number of patients with first and second episodes. This size imbalance can hinder the accuracy and reliability of the analyses in assessing the relationships between the number of episodes and various characteristics, including anxiety.

## Conclusions

In patients with RE of BPPV, compared to patients with the FE, trait anxiety was higher throughout the study, and state anxiety was higher in the second meeting. In light of the current study’s findings, we recommend assessing trait anxiety levels in patients with recurrent BPPV and referral to suitable treatments if necessary.

## Data Availability

The raw data supporting the conclusions of this article will be made available by the authors without undue reservation.

## References

[1] Agrawal Y, Ward K, Minor B (2013). Vestibular dysfunction: prevalence, impact and need for targeted treatment. J Vestib Res.

[2] Bhattacharyya N, Gubbels P, Schwartz R, Edlow A, El-Kashlan H, Fife T, Holmberg M, Mahoney K, Hollingsworth B, Roberts R, Seidman D, Steiner P, Do T, Voelker J, Waguespack W, Corrigan D (2017). Clinical practice guideline: benign paroxysmal positional vertigo (update). Otolaryngol Head Neck Surg.

[3] Kim J, Park H, Kim S (2020). Update on benign paroxysmal positional vertigo. J Neurol.

[4] Li S, Wang Z, Liu Y, Cao J, Zheng H, Jing Y, Han L, Ma X, Xia R, Yu L (2022). Risk factors for the recurrence of benign paroxysmal positional vertigo: a systematic review and meta-analysis. Ear Nose Throat J.

[5] Shu Y, Liao N, Fang F, Shi Q, Yan N, Hu Y (2023). The relationship between psychological conditions and recurrence of benign paroxysmal positional vertigo: a retrospective cohort study. BMC Neurol.

[6] Casani A, Pietro, Gufoni M (2023). Recurring benign paroxysmal positional vertigo after successful canalith repositioning manoeuvers. Acta Otorhinolaryngol Italica.

[7] Chen J, Zhang S, Cui K, Liu C (2021). Risk factors for benign paroxysmal positional vertigo recurrence: a systematic review and meta-analysis. J Neurol.

[8] Pollak L, Kushnir M, Goldberg HS (2011). Physical inactivity as a contributing factor for onset of idiopathic benign paroxysmal positional vertigo. Acta Otolaryngol.

[9] Picciotti PM, Lucidi D, De Corso E, Meucci D, Sergi B, Paludetti G (2016). Comorbidities and recurrence of benign paroxysmal positional vertigo: personal experience. Int J Audiol.

[10] Özdilek A, Dikmen Y, Acar E, Aksoy A, Korkut N (2019). Determination of anxiety, health anxiety and somatosensory amplification levels in individuals with benign paroxysmal positional vertigo. J Int Adv Otol.

[11] Yeo BSY, Toh EMS, Lim NE, Lee RS, Ho RCM, Tam WWS, Ngo RYS (2024). Association of benign paroxysmal positional vertigo with depression and anxiety—a systematic review and meta-analysis. Laryngoscope.

[12] Chen J, Chang H, Hu Y, Tu S, Lu T, Chen M, Shen C (2016). Increased risk of benign paroxysmal positional vertigo in patients with anxiety disorders: a nationwide population-based retrospective cohort study. BMC Psychiatry.

[13] Kalderon L, Chaimoff M, Katz-Leurer M (2022). The distinction between state and trait anxiety levels in patients with BPPV in comparison with healthy controls. Front Psychol.

[14] Spielberger D, Gorsuch L, Lushene R, Vagg R, Jacobs A (1968) State–trait-anxiety-inventory for adults self-evaluation questionnaire STAI form Y-1 nad form Y-2. p. 5

[15] Kaplan DM, Friger M, Racover NK, Peleg A, Kraus M, Puterman M (2010). The Hebrew dizziness handicap inventory. Harefuah.

[16] Julian J (2011). Measures of anxiety: state–trait anxiety inventory (STAI), beck anxiety inventory (BAI), and hospital anxiety and depression scale-anxiety (HADS-A). Arthritis Care Res.

[17] Keinan G, Zeidner M (1987). Effects of decisional control on state anxiety and achievement. Pers Individ Differ.

[18] Jacobson P, Newman W (1990). The development of the dizziness handicap inventory. Arch Otolaryngol Head Neck Surg.

[19] Mutlu B, Serbetcioglu B (2013). Discussion of the dizziness handicap inventory. J Vestib Res.

[20] Fielder H, Denholm W, Lyons A, Fielder P (1996). Measurement of health status in patients with vertigo. Clin Otolaryngol.

[21] Godin G, Shephard J (1985). A simple method to assess exercise behavior in the community. Can J Appl Sport Sci.

[22] Kahraman S, Arli C, Copoglu S, Kokacya H, Colak S (2017). The evaluation of anxiety and panic agarophobia scores in patients with benign paroxysmal positional vertigo on initial presentation and at the follow-up visit. Acta-laryngol.

[23] Gunes A, Yuzbasioglu Y (2019). Effects of treatment on anxiety levels among patients with benign paroxysmal positional vertigo. Eur Arch Otorhinolaryngol.

[24] Wei W, Sayyid N, Ma X, Wang T, Dong Y (2018). Presence of anxiety and depression symptoms affects the first time treatment efficacy and recurrence of benign paroxysmal positional vertigo. Front Neurol.

[25] Balaban CD, Thayer JF (2001). Neurological bases for balance-anxiety links. J Anxiety Disord.

[26] Kozak H, Dundar A, Uca U, Uguz F, Turgut K, Altas M, Tekin G, Aziz K (2016). Anxiety, mood, and personality disorders in patients with benign paroxysmal positional vertigo. Noro Psikiyatri Arsivi.

[27] Chiarella G, Russo D, Monzani F, Petrolo C, Fattori B, Pasqualetti G, Cassandro E, Costante G (2017). Hashimoto thyroiditis and vestibular dysfunction. Endocr Pract.

[28] Tricarico L, Di Cesare T, Galli J, Fetoni R, Paludetti G, Picciotti M (2022). Benign paroxysmal positional vertigo: is hypothyroidism a risk factor for recurrence?. Acta Otorhinol.

